# Features of a Patient Portal for Blood Test Results and Patient Health Engagement: Web-Based Pre-Post Experiment

**DOI:** 10.2196/15798

**Published:** 2020-07-20

**Authors:** Bas Struikman, Nadine Bol, Annelijn Goedhart, Julia C M van Weert, Esther Talboom-Kamp, Sanne van Delft, Anne E M Brabers, Liset van Dijk

**Affiliations:** 1 Nivel (Netherlands Institute for Health Services Research) Utrecht Netherlands; 2 Department of Communication and Cognition Tilburg School of Humanities and Digital Sciences Tilburg University Tilburg Netherlands; 3 Department of Communication Science Amsterdam School of Communication Research University of Amsterdam Amsterdam Netherlands; 4 Saltro, Diagnostics Center for Health Care Utrecht Netherlands; 5 Department National eHealth Living Lab Leiden University Leiden Netherlands; 6 Department of PharmacoTherapy, -Epidemiology & -Economics Groningen Research Institute of Pharmacy, Faculty of Science and Engineering University of Groningen Groningen Netherlands

**Keywords:** online patient portals, patient health engagement, blood testing, blood test results, consumer panel, visualization, patient information

## Abstract

**Background:**

The use of patient portals for presenting health-related patient data, such as blood test results, is becoming increasingly important in health practices. Patient portals have the potential to enhance patient health engagement, but content might be misinterpreted.

**Objective:**

This study aimed to discover whether the way of presenting blood test outcomes in an electronic patient portal is associated with patient health engagement and whether this varies across different blood test outcomes.

**Methods:**

A 2x3 between-subjects experiment was conducted among members of the Nivel Dutch Health Care Consumer Panel. All participants read a scenario in which they were asked to imagine themselves receiving blood test results. These results differed in terms of the presented blood values (ie, normal vs partially abnormal vs all abnormal) as well as in terms of whether the results were accompanied with explanatory text and visualization. Patient health engagement was measured both before (T0) and after (T1) participants were exposed to their fictive blood test results.

**Results:**

A total 487 of 900 invited members responded (response rate 54%), of whom 50.3% (245/487) were female. The average age of the participants was 52.82 years (SD 15.41 years). Patient health engagement saw either a significant decrease or a nonsignificant difference in the experimental groups after viewing the blood test results. The mean difference was smaller in the groups that received blood test results with additional text and visualization (mean_T0_ 5.33, SE 0.08; mean_T1_ 5.14, SE 0.09; mean difference 0.19, SE 0.08, *P*=.02) compared with groups that received blood test results without explanatory text and visualization (mean_T0_ 5.19, SE 0.08; mean_T1_ 4.55, SE 0.09; mean difference 0.64, SE 0.08, *P*<.001). Adding text and visualization, in particular, reduced the decline in patient health engagement in participants who received normal results or mixed results (ie, combination of normal and abnormal results).

**Conclusions:**

Adding text and visualization features can attenuate the decrease in patient health engagement in participants who receive outcomes of a blood test via a patient portal, particularly when blood test results are (partly) normal. This suggests that explanatory text and visualization can be reassuring. Future research is warranted to determine whether these results can be generalized to a patient population who receive their actual blood test results.

## Introduction

Patient health engagement (PHE) can play an important role in personal health and can improve adherence and satisfaction with received care [1,2]. From a holistic perspective, PHE can be defined as a multidimensional process, including elements of cognition (thinking), emotion (feeling), and behavior (acting) of a patient towards his or her role in health management [3]. Patients’ engagement in their health management is dependent on the stability of these dimensions. A lack of understanding of their position in health care can immobilize patients, making them indecisive or apathetic, while a better understanding makes them more active [3]. Engaged patients who want to be informed about and take an active role in their own health care are more likely to show healthy behaviors, have better self-management, and achieve better health-related outcomes [2,4]. Vice versa, lower PHE has been related to preventable deaths and unnecessary costs [5]. One stage where PHE is important is the stage of diagnosis [6]. When patients take an active role by asking questions and voicing their opinions, they enhance their role in their health management and increase their empowerment [7]. An important part of the stage of the diagnostic process is the information that becomes available from blood tests. However, this information can cause insecurity and uncertainty for the patient. This can negatively impact PHE as patients can become emotionally destabilized by the confusion or impact of the test results [7]. This risk is bigger when patients find the blood test values difficult to interpret [8].

Currently, electronic patient portals are increasingly offered by health professionals to communicate blood test results to the patient. Even though these portals are not optimally used yet [9], patient portals are becoming increasingly important in the health care sector and valued by both patients and practitioners [10]. Patient portals have been designed to encourage patient involvement. Yet, the way the content is presented in a portal and the way the patient interprets such content impact the overall usefulness of the information [10]. To prevent unnecessary anxiety, blood test results must be presented properly to patients [7] and needs to be done in such a way that it does not jeopardize PHE. In health practices, blood test results in a standard portal are usually communicated by giving the quantitative results (ie, the patient’s value) plus reference values (ie, a range that expresses the normal values for that test). Added features such as text, symbols, or visuals to help interpret blood test results are usually not provided. However, problems have been reported about the usage of such a basic portal. For instance, the limited amount of information makes interpretation of results complex, which makes the blood test results only useful for patients with high health literacy [11]. Furthermore, misinterpretation has led to patients underestimating the severity of blood test outcomes [12].

One of the main problems is that patient portals often rely on numerals to purvey information. This is a concern for people with low numeracy skills (ie, people who lack the ability to use and draw meaning from numbers). People with low numeracy skills have shown more difficulties with identifying out-of-range test results [13]. This is likely due to the unfamiliar abbreviations, unfamiliar units, and little guidance as to whether higher numbers represent more positive or negative outcomes. This problem increases when larger sets of values are displayed at once [13]. These findings raise concerns for patient safety. Arguments for keeping this type of portal can be that people, who searched online for information, found that websites often have too much information for them to comprehend [14]. Furthermore, detailed information was found to be overwhelming for certain patients [15]. Thus, a patient portal can be useful to provide patients with individualized information (in this case, blood test results) without irrelevant information. However, this information has to be presented in a comprehensible way.

There are good reasons to believe that textual explanations with explanatory visual aids can benefit patients [16], both online and offline. For example, visualization to communicate different levels of driving risks (ie, yellow, orange, and red bars) provided good insight into the risk level of driving while using a specific medicine [17]. When risk communication is done using well-designed visual aids, information through patient portals could be received with fewer problems and enhance consent to further treatment [18]. Infographics have also been shown to be of added value in delivering complex information [19], and graphs helped a third of patients with lower numerical skills in transferring risk information [20]. When people do not understand the information, they will often use their “gut” feeling to make decisions about uncertain situations [21]. This can be a problem for PHE, as this feeling can make a patient more passive in their health management. Little is known about how presenting blood test values in a patient portal can influence PHE. The aim of this study was to explore whether the way blood test outcomes are presented in a patient portal is associated with PHE and whether this varies across different blood test outcome combinations. Exploratory research can be a crucial step to further develop scientific knowledge by laying groundwork for research topics that do not yet have a strong basis for hypotheses [22]. An experiment was conducted to systematically test the effect of different blood test results presented in a basic patient portal as well as in a patient portal in which text and visualization was added to the standard way of presenting blood test results.

## Methods

### Portal

The basis of our study was the comparison of two portals. Both portals communicated blood test results accompanied by reference values (ie, the range that expresses the normal values for that test). These types of portals are most often used in Dutch clinical practice, which was the setting of our study. The first portal was a fictive basic portal only providing the patient’s blood values with the corresponding reference categories. The second portal was based on a more sophisticated portal as developed by Saltro, one of the largest diagnostic centers in the Netherlands. The Saltro portal adds two main features compared to the first portal: (1) textual understandable information explaining the test and its outcomes and the action the patient can take afterwards and (2) visual support by using traffic light colors to indicate whether the outcome is within the normal range. The content of this portal was cocreated with health care professionals, communication experts, and patients. The text was written to be understandable for the majority of people. The level of health literacy of the results information has been estimated at communication level 1B on the scales of the Common European Framework of Reference for Languages. Furthermore, the content is frequently evaluated by patients and adapted according to their recommendations.

### Design and Procedure

A 2x3 between-subjects experimental design was employed to test the effects of the blood test results outcome and the addition of explanatory text and visualization on PHE. Participants were so-called analog or simulated patients (ie, people who imagine themselves being in a hypothetical health care situation) presented with a hypothetical case (see Textbox 1). We opted for analog patients for two reasons. First, since we manipulated the blood test outcomes, it was unethical to use “real” patients receiving their own blood test results. Second, analog patients can be used in study designs such as ours, based on a meta-analysis that demonstrated the validity of using analog patients by showing insignificant discrepancies between the perceptions of analog patients and clinical patients [23]. The hypothetical case was identical for every participant (Textbox 1). Fatigue was chosen as a health problem as it is easy to relate to. In short, the case description stated that participants had to envision they have been tired for a couple of months now and it is not getting any better. We chose an excessive form of fatigue in order to arouse feelings of fear or worry in the participant. They went to a general practitioner who ordered some blood tests. The outcomes were to be communicated through a web portal.

We tested the two types of portals (ie, with vs without explanatory text and visualization), within which we distinguished three possible outcomes of the blood tests: all values within range (normal), partially deviating values (partially abnormal), and all deviating values (all abnormal). Table 1 shows the 6 conditions that were tested. The outcomes per blood value group were identical. Participants were randomly assigned to 1 of the 6 experimental conditions without knowing about the other 5 conditions.

**Fictional case presented to each participant, translated from Dutch to English.**

You have been tired for a couple of months now, and it does not get any better. No matter how much you sleep, you remain tired. You even fell asleep at work once. You do not feel tense or stressed. It does bother you that the tiredness does not go away. You decide to visit your general practitioner (GP) to describe your symptoms to better understand/get a grip on your situation. The GP asks if you have any thoughts on the cause of the tiredness. You have no clue. Your private life is fine, you have never been this tired before, your diet is healthy, and you do not smoke or use medication. You have become worried about the situation; what is going on? The GP suggests to first run some blood tests before making any decisions. The GP explains that she wants to measure 3 types of blood values: tests for an underactive thyroid (thyroid stimulating hormone), anemia (hemoglobin), and a deficit of vitamin D. In the morning, your blood gets drawn, and you are told that your results are available at your convenience on the website of your GP through a patient portal the same day.

**Table 1:** The 6 groups of the 2x3 design.

Portal type	All normal values	Partially abnormal values	All abnormal values
No features	“Green basic”	“Partial orange basic”	“Orange basic’”
Added features^a^	“Green Saltro”	“Partial orange Saltro”	“Orange Saltro”

^a^Explanatory text with added visuals (see Figure 1).

**Figure 1:**
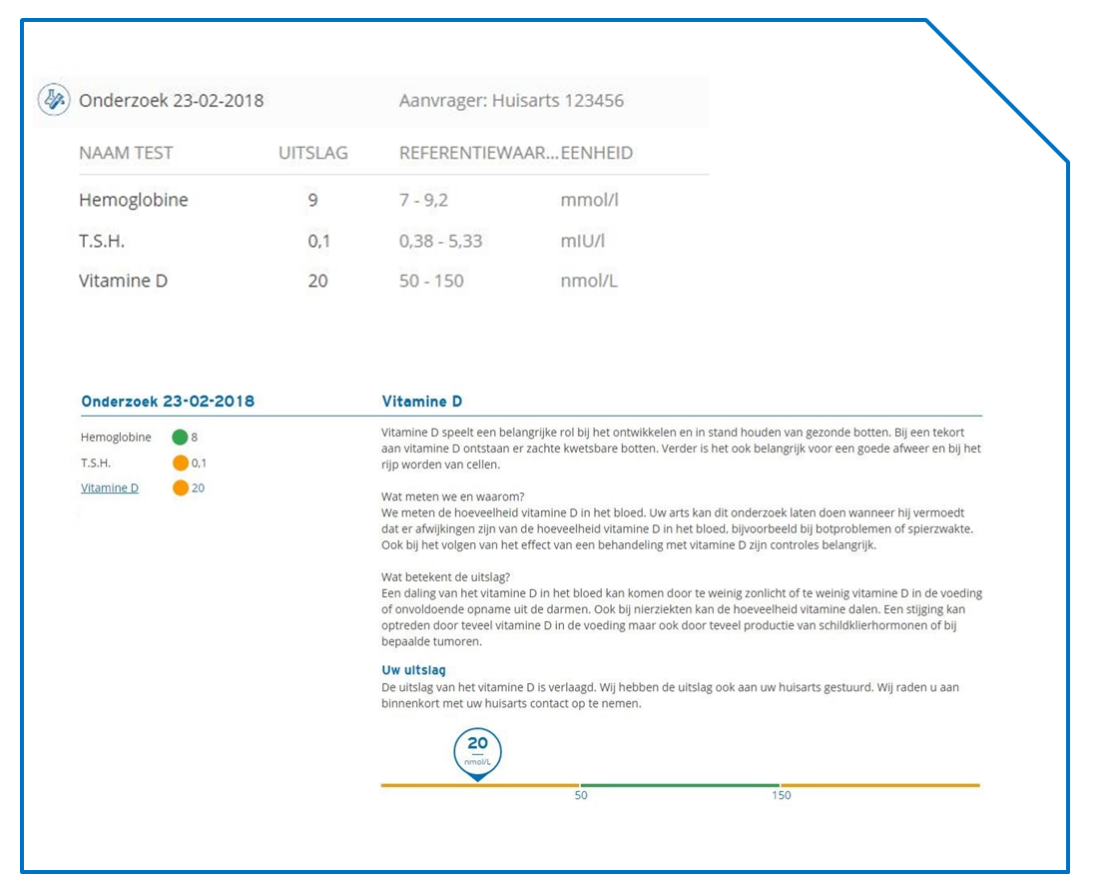
Basic portal (top) and Saltro portal (bottom), both displaying the same partially abnormal results. All text is in Dutch and is shown in a similar fashion for hemoglobin and thyroid stimulating hormone.

### Stimulus Materials

The Saltro portal adds explanatory text that gives an explanation about which function the substance has in the body and what exactly is being measured, while showing a bar that presents the patient’s blood value in an orange (abnormal) or green (normal) range. A marker shows where the patient’s blood test result falls within the range (see Figure 1). The text also offers an explanation on why measuring this particular substance is important, what it could mean if a value is below or above the normal range, and an indication of possible causes for the abnormal result. The text encourages the patient to contact their physician for further questions. The text ends with a conclusion stating whether the blood test result of the patient is below, above, or within the normal value range. Participants were exposed to 3 blood tests: hemoglobin, thyroid stimulating hormone, and vitamin D. The total word counts of the explanatory text were 271 for hemoglobin, 175 for thyroid stimulating hormone, and 194 for vitamin D.

### Participants

We approached a sample of 900 participants of the Dutch Health Care Consumer Panel to complete an online questionnaire. This panel aims to measure opinions on and knowledge of health care as well as the expectations of and experiences with health care among a cross-section of the Dutch population [24]. The Dutch Health Care Consumer Panel is an access panel consisting of people who have agreed to answer questionnaires on a regular basis. Some sociodemographic details of the participants are known, such as age, gender, and highest level of education. In June 2018, the Consumer Panel consisted of about 12,000 people. To be included in the panel, a respondent has to be 18 years or older. All participants typically are asked to complete a questionnaire 3-4 times a year. Participants have complete liberty to decide whether to only answer certain questions or to participate at all. The technical functionality of the questionnaires is tested by the researcher before sending out the questionnaire. Resigning from the panel can be done at any time. People cannot sign up for the panel on their own initiative, and as such, we used a closed survey. The response rate for this study was stimulated by sending two electronic reminders to panel members who had not responded yet. The closing date of the questionnaire was at the end of May 2018. Panel members did not receive financial compensation.

Data were analyzed anonymously and processed according to the privacy policy of the Dutch Healthcare Consumer Panel, which complies with the General Data Protection Regulation. According to Dutch legislation, there is no legal requirement to obtain informed consent nor approval by a medical ethics committee for conducting research through the panel [25]. Privacy regulation is available.

### Measurements

We used the original 9-item version of the PHE scale as the starting point for our measurement [3]. The PHE scale defines patient health engagement as a multidimensional process, including elements of emotion (feeling), cognition (thinking), and behavior (acting) towards his or her role in health management. Of the 9 original items, 5 items were considered appropriate for this experiment. For 2 items, the answer categories were slightly adapted to make it more applicable to the case in our study. Of the original PHE scale, 4 items were considered irrelevant for this study, as they specified feelings about the illness itself and could not be used due to the fictional nature of the case study. Participants were asked to indicate the extent to which the 7-point semantic differential scales applied to them. The items were: “I feel in blackout” versus “I feel positive,” “I feel dazed” versus “I feel serene,” “I can’t understand what happened to me” versus “I understand what this situation means to me,” “I feel totally messed up” versus “I know what to do in this situation,” and “I let others take care of me” versus “I can autonomously manage my medical regime.” The participants filled out the 5-item version of the PHE scale both before (T0) and after (T1) exposure to the patient portal. All 5 items were averaged into one mean scale of PHE at T0 (Cronbach α=.90) and T1 (Cronbach α=.92). As individual items showed similar trends to those of the overall items, we did not analyze those separately. A higher score on the mean scale means a higher PHE. Because T0 was the baseline measurement (after exposure to the case scenario but before the patient portal with the blood test results), it could be expected that the health engagement score would not increase after exposure to the portal with the blood test results, but either remain the same or decrease, depending on the extent to which the blood test results would evoke elements of emotion, cognition, or behavior that could hinder patients’ health engagement. The reason for this is that when the results are abnormal, the level of PHE is expected to be lower after receiving the test. Also, for those who receive normal results, an increase is not obvious because it means that the cause for the long-term tiredness is not clear.

### Statistical Analysis

For the randomization check, we examined whether participant characteristics were equally divided across experimental conditions using *F* tests and chi-square tests. For age, education, and health status, we conducted 3 two-way analyses of variance with differences between “text/visualization added” and between “outcome of blood test results” as the between-subjects factors and age, education, and health status as the dependent variables. Health status was measured with a single question asking how the participants would rate their own health (1=excellent, 5=poor). For gender, we conducted a chi-square test with the combined experimental factors as one variable and gender as the other variable.

For the main analysis, a mixed analysis of variance was conducted with “text/visualization added” and “outcome of blood test results” as the between-subjects factors and “pretest measure of patient health engagement” versus “posttest measure of patient health engagement” as the within-subjects factor or repeated measure. Main effects of the between-subjects factors (ie, “text/visualization added” and “outcome of blood test results”) and within-subjects factor (ie, “pretest measure of patient health engagement” versus “posttest measure of patient health engagement”) were calculated. Furthermore, the interaction effect between the between-subjects factors (ie, “text/visualization added” × “outcome of blood test results”), two-way interaction effects between the between-subjects and within-subjects factors (ie, “text/visualization added” × “pretest vs posttest” and “outcome of blood test results” × “pretest vs posttest”), and three-way interaction effect between factors (ie, “text/visualization added” × “type of blood test results” × “pretest vs posttest”) were assessed. Simple effects analyses were conducted in case of significant interactions between variables.

## Results

### Sample Characteristics

Of the 519 participants that started the survey, 487 had sufficiently complete data (ie, ≥60% of the PHE measure completed). Table 2 shows the descriptive statistics of these 487 participants. Participants were, on average, 53 years old (mean 52.82 years, SD 15.41 years) and reported to be in good to very good health (mean 2.65, SD 0.87). About half of the participants were female (245/487, 50.3%), and most people had completed a middle level (219/487, 45.8%) or higher level (222/487, 46.4%) of education. The randomization check presented no significant differences between the 6 experimental conditions with respect to gender (χ²_5_=7.52, *P*=.19), age, (*F*_2,472_=0.32, *P*=.73, η²=.00), education (*F*_2,472_=0.00, *P*=1.00, η²=.00), and health status (*F*_2,472_=0.13, *P*=.88, η²=.00). No control variables were included in the analyses.

**Table 2:** Sample characteristics (N=487).

Characteristics			Values
Age (years), mean (SD; range)			52.82 (15.41; 24-90)
**Gender, n (%)**			
	Male	242 (49.7)	
	Female	245 (50.3)	
**Education^a^, n (%)**			
	Low	37 (7.7)	
	Middle	219 (45.8)	
	High	222 (46.4)	
Health status^b^, mean (SD; range)			2.65 (0.87; 1-5)

^a^Values for education do not add up to 487 due to missing data.

^b^Self-reported health status (how would you rate your own health) ranges from 1 = excellent to 5 = poor.

### Effects of the Outcome of the Blood Test Results

We found that the outcome of the blood test results, regardless of the portal design, impacted PHE after exposure to the blood test results (*F*_2,481_=6.65, *P*<.001, η_p_²=.03). Receiving normal blood test results did not significantly decrease PHE (mean_T0_ 5.27, SE 0.10; mean_T1_ 5.10, SE 0.11; mean difference 0.17, SE 0.09, *P*=.07), but PHE significantly decreased after receiving abnormal blood test results (mean_T0_ 5.35, SE 0.10; mean_T1_ 4.69, SE 0.11; mean difference 0.66, SE 0.10, *P*<.001) or partially abnormal blood test results (mean_T0_ 5.16, SE 0.10; mean_T1_ 4.75, SE 0.11; mean difference 0.41, SE 0.10, *P*<.001).

### Effect of Textual and Visual Explanation

Furthermore, adding text and visualization that explained the blood test results impacted PHE after exposure to blood test results (*F*_1,481_=16.83, *P*<.001, η_p_²=.03). Although receiving blood test results with additional text and visualization significantly decreased PHE after receiving the results (mean_T0_ 5.33, SE 0.08; mean_T1_ 5.14, SE 0.09; mean difference 0.19, SE 0.08, *P*=.02), this decrease was significantly larger when blood test results were presented without explanatory text and visualization (mean_T0_ 5.19, SE 0.08; mean_T1_ 4.55, SE 0.09; mean difference 0.64, SE 0.08, *P*<.001).

### Interaction Between the Type of Outcome of the Blood Test Result and Textual and Visual Explanation

A significant interaction effect between the outcome of the blood test results and the addition of explanatory text and visualization revealed a nuanced insight into how PHE develops after being exposed to blood test results. It showed that, for all outcomes of blood test results, a lack of explanatory text and visualization decreased PHE after being exposed to the results (*F*_2,481_=3.83, *P*=.02, η_p_²=.02). More specifically, this decline occurred for normal blood test results (mean_T0_ 5.02, SE 0.14; mean_T1_ 4.73, SE 0.16; mean difference 0.29, SE 0.13, *P*=.03), abnormal blood test results (mean_T0_ 5.39, SE 0.14; mean_T1_ 4.61, SE 0.09; mean difference 0.77, SE 0.13, *P*<.001), and partially abnormal results (mean_T0_ 5.16, SE 0.15; mean_T1_ 4.31, SE 0.16; mean difference 0.85, SE 0.14, *P*<.001). However, when explanatory text and visualization were added to the blood test results, we found no significant decline in PHE for normal (mean_T0_ 5.51, SE 0.14; mean_T1_ 5.46, SE 0.16; mean difference 0.05, SE 0.13, *P*=.71) and partially abnormal (mean_T0_ 5.16, SE 0.14; mean_T1_ 5.19, SE 0.16; mean difference –0.03, SE 0.13, *P*=.81) blood test results. Yet, in the case of abnormal blood test results, even with explanatory text and visualization, PHE significantly decreased after receiving the blood test results (mean_T0_ 5.31, SE 0.15; mean_T1_ 4.73, SE 0.17; mean difference 0.54, SE 0.14, *P*<.001). These findings are presented in Table 3.

**Table 3:** Descriptive statistics for patient health engagement (PHE) before (T0) and after (T1) exposure to the patient portal across the experimental conditions (N=487).

Experimental conditions		n	PHE at T0, mean (SE)	PHE at T1, mean (SE)	*P* value
**Normal blood test results**					
	Without text	83	5.02 (.14)	4.73 (.16)	.03
	With text	82	5.51 (.14)	5.46 (.16)	.71
**Partially abnormal blood test results**					
	Without text	87	5.16 (.15)	4.31 (.16)	<.001
	With text	74	5.16 (.14)	5.19 (.16)	.81
**Abnormal blood test results**					
	Without text	78	5.39 (.14)	4.61 (.09)	<.001
	With text	83	5.31 (.15)	4.73 (.17)	<.001

## Discussion

### Principal Findings

The aim of this study was to discover whether the way in which blood test outcomes are presented in a patient portal is associated with PHE and whether this varies across different blood test outcomes. Adding textual and visual explanations to blood test results minimizes the decline in PHE when receiving blood test results in an electronic patient portal. When presenting blood test results through an existing patient portal, the group that received explanatory text and visualization in addition to their results experienced less of a decline in PHE than the group without these features. This was particularly true for patients who received normal and partially abnormal results (ie, combination of normal and abnormal results). For patients who received abnormal results for all three blood tests, health engagement significantly decreased independent of whether explanatory text and visualization were added. It can be concluded that adding text and visualization to a patient portal can attenuate PHE and therefore involve patients more in their health management, but only when blood test results are normal or partially abnormal.

### Comparison With Prior Work

The focus of our study was to gain insight into how providing explanatory text and visualization, when presenting blood test results via a patient portal, influences PHE. While previous research has shown that patients are generally satisfied with the use of a patient portal to check their blood test results [16], there are also concerns with such portals.

One recurring concern with patient portals is the fear of the misinterpretation of results [9,10]. For example, in a study by Korngiebel et al [26], clinicians’ main concern was that providing blood test results to patients without explanation could lead to confusion due to the sensitive and complicated nature of the test results. Especially divergent results would have to be carefully shown. Although our results cannot confirm if the nature of the feelings of our participants are due to confusion, it does show signs of fear, as lower PHE is associated with greater emotional immobilization. Participants who received normal or partially abnormal results in the “standard” way showed a significant decline in PHE, while health engagement in patients who received explanatory information and visual support remained at the same level as at baseline.

In this study, we did not distinguish between the added value of explanatory text only and the added value of visualization only. Hence, we do not know which of these two features was the “active ingredient” or whether the combination of the features caused the effectiveness of the patient portal. In two previous studies, information provision in a patient portal was evaluated in isolation. In the first study, visual support using a color scheme to differentiate between normal results and divergent results was positively evaluated by elderly patients [27]. In the second study, veteran patients did not have extra text available but instead were given a search bar to find relevant health information themselves [28]. The veterans evaluated the search bar positively. Both these studies based the evaluation of the patient portal on opinions, whereas our study measured effectiveness in terms of PHE.

In addition, our study measured PHE using a questionnaire. Previous studies have used different ways to measure (patient) engagement. To illustrate, Phelps et al [29] defined engagement in terms of portal usage and measured it by monitoring the number of logins. They found an increase in logins, for instance shortly before meeting a physician, and results were therefore seen as positive. We used a different operationalization of engagement, including emotions and cognitions that test results can evoke, which resulted in a more nuanced picture of how people can respond upon being confronted with blood test results. The explanatory text and visualization had a positive influence on PHE, such that it reduced the decline in engagement after being exposed to blood test results. It did not improve PHE compared to the baseline measure. However, since we were studying how people react upon exposure to (potential) risk information, it seems to make sense to aim for a reduction of negative reactions rather than for an increase of positive reactions.

### Strengths and Limitations

A strength of our study is that it systematically tested the impact of patient portal design on PHE in a highly controlled experimental setting. Although patient portal designs have been introduced to patients, these have not been studied yet in terms of how their design can impact PHE. In addition, our measurement of PHE gave a broad depiction of PHE through patients’ feelings, thoughts, and behaviors. Therefore, our study design gives this study a good foundation for its conclusions and implications.

There were also some limitations to our study. The first one is the potential bias in our sample. Although the sample was randomly selected from the Nivel Consumer Panel, which represented a relatively diverse group of patients, the participants of this panel are people who agreed to fill out questionnaires about health and health care. This means that there is good reason to believe that a majority of the people have, at least to some extent, affinity with their personal health (and health care). Furthermore, it can be assumed that the average panel user has a higher health literacy than normal. Health literacy entails the capability of obtaining, processing, and understanding information about health and health services [30]. If this assumption holds true, it is relevant to consider that affinity with health care might coincide with a better understanding and processing of health care information. Thus, extra information might not be as vital to them as to those with lower health literacy. In that sense, our sample resembled the target group of the portal with added text, as both our questionnaire and the portal require some level of functional literacy.

The necessity for participants to empathize with a fictional case was another limitation. Actual patients, for whom results might indeed have an impact on their life, might have reacted differently to the results of the blood test as they might be expected to be more emotionally involved. Based on the meta-analysis by Van Vliet et al [23], we do not expect that the results in actual patients will be weaker than in the current sample of analog patients, and they might even be stronger in actual involved patients. Thus, a replication of our study with actual patients would be of added value. Moreover, we used the original 9-item PHE scale [3] as the starting point for our measurement while rephrasing and shortening it for the purpose of our study, resulting in a scale with high internal consistency. The reason for doing this was that there is no scale available that is validated for the case in our study (tiredness).

Another possible limitation is the specific design of our portal. Our results could not cover all portal designs, so the scope of the study may be limited and not generalizable to other patient portals. For instance, the colors of our “complementary” portal design could have influenced the feelings of the participants. Research has shown that the color red can evoke feelings with patients [31]. The color orange might cause an unnecessary reaction as well, since it can be associated with danger or a sign for increased attention. It could therefore be that a participant who viewed a full or partially orange result were more anxious, although an abnormal result does not automatically mean a danger to your health or the need for immediate medical intervention. Future research can address the consequences by implementing more neutral colors. This way, a patient’s anxiety can be ascribed to the conclusion of the blood test result and not stimulation by a bright color. Finally, as mentioned before, our existing portal had two features: additional text for explanation and added visualization for understanding the blood levels. The downside of this is that a distinction cannot be made between the impact of the text and that of the visualization. If electronic patient portals are to be improved, it is necessary to understand if the limiting impact on the decline in PHE is due to either one of the features or perhaps the interaction between them.

### Clinical and Research Implications

Our study has provided insight into how portal design can benefit PHE. Our results suggest that institutions that use a patient portal for blood test results might want to consider adding complementary information and visual support. Further testing of patient portal features is recommended, but institutions would do well to start adding visualization and textual support to their portals as this can benefit PHE. Adding text and visualization that explain the blood values and the implications of an abnormal result might support their patients in their health management. It might lead to patients who are more involved in their diagnosis and treatment, which could lead to, for instance, patients being more comfortable voicing concerns or asking questions. As mentioned, there is a possibility that our sample consisted of participants with an above-average health literacy. Therefore, future research could focus on patients with below-average health literacy who might need a different portal design. Yet, more research among actual patients is needed to test the portal with patients who are personally involved and therefore more concerned about the outcomes of blood test results. For example, in case a fictional study is chosen, this fictional case could include a disease with a higher emotional impact and familiarity, such as cancer. For such a disease, a more intense reaction could occur while viewing blood test results, such as relief or anxiety, which in turn could have a stronger effect on PHE. However, ethical considerations should be taken into account, as participants often do not know that they are being confronted with such an emotional case. A warning up front for the participant might be considered if the need for a more emotional investment is deemed necessary. Lastly, we used an adaptive version of the validated, original PHE scale. By deleting 4 items, it is possible that it did not cover the full domain of PHE (ie, did not measure the domains of feelings, thoughts, and behaviors as fully as the original scale).

### Conclusion

Patient portals have been designed to improve patient involvement. When blood test results are communicated to patients, it can negatively affect their PHE and consequently their involvement in their health management. However, when these outcomes are supported by explanatory text and visualization to help interpret the outcomes, the decrease in PHE can be attenuated, especially when test results are partially normal. As receiving test results can cause feelings of uncertainty in patients, which can lead to lower PHE, our results suggest that explanatory text with visualization can cause feelings of relief in patients. Future research should focus on repeating the experiment with actual patients who receive their own blood test results to test whether the results hold in more ecologically valid settings.

## Abbreviations

GP: general practitioner
PHE: patient health engagement
